# Vapor pressure deficit normalizes diurnal photochemical reflectance index for repeatable drought phenotyping of *Prunus* rootstocks in Mediterranean climates

**DOI:** 10.3389/fpls.2026.1878667

**Published:** 2026-10-21

**Authors:** Guillermo Toro, Paula Pimentel, Ariel Salvatierra, Ismael Opazo, Félix Estrada, Gonzalo A. Díaz, José Cares, Claudia Moggia, Gustavo A. Lobos

**Affiliations:** 1 Centro de Estudios Avanzados en Fruticultura (CEAF), Rengo, Chile; 2 Instituto de Investigaciones Agropecuarias (INIA), Rayentué, Rengo, Chile; 3 Laboratory of Fruit Pathology, Facultad de Ciencias Agrarias, Universidad de Talca, Talca, Chile; 4 Plant Breeding and Phenomics Center, Facultad de Ciencias Agrarias, Universidad de Talca, Talca, Chile

**Keywords:** diurnal dynamics, drought, gas exchange, phenomics approaches, phenotyping

## Abstract

Breeding programs need field-ready traits to compare rootstock drought performance under fluctuating atmospheric demand, particularly in Mediterranean climates where vapor pressure deficit (VPD) can increase abruptly during the day and confound midday “snapshot” screening. We present a VPD-based method to normalize and decompose diurnal dynamics of the photochemical reflectance index (PRI) into repeatable components. Six *Prunus* rootstocks genotypes were grown under well-watered (WW) and water-deficit (WD; 40% of field capacity) conditions. Leaf PRI was measured hourly (10:00–18:00) on three days spanning VPDmax ≈ 4.9–3.3 kPa, while midday gas exchange and leaf water potential were measured on two days. PRI relationships with incident radiation were weak and variable across genotypes and dates, whereas PRI–VPD trajectories were structured and consistently separated WW and WD conditions. For each genotype, date, and water-regime, PRI–VPD regressions were fitted and decomposed into (i) basal PRI at a common reference VPD (VPDref = 2.0 kPa), (ii) the diurnal PRI–VPD slope, and (iii) WW–WD separation at VPDref. Basal PRI and WW–WD separation produced stable genotype rankings across days (Spearman ρ ≈ 0.8–0.9). Across genotypes, water-deficit–induced changes in PRI (WD relative to WW) tracked changes in stomatal conductance (gs) and transpiration (E). This VPD-normalized decomposition provides a scalable optical trait to quantify diurnal stress signatures for drought phenotyping in Mediterranean environments.

## 1 Introduction

Drought and heat are intensifying due to climate change (Vogel et al., 2019; Pareek et al., 2020), emphasizing the need for reliable high-throughput phenotyping traits. In stone-fruit systems, rootstocks affect water relations, stomatal regulation, and drought tolerance, showing significant genotypic variation for traits linked to transpiration control and water-use efficiency (Hancock et al., 2008; Jiménez et al., 2013; Gainza et al., 2015; Opazo et al., 2019, 2020). However, breeding-oriented screening typically relies on single-time-point measurements near solar noon (Medrano et al., 2018; Leakey et al., 2019; Lobos et al., 2022), which may miss genotype differences in how physiology is regulated throughout the day as atmospheric conditions fluctuate.

*Prunus* rootstocks strongly modulate scion water relations, stomatal regulation and drought tolerance, and rootstock breeding increasingly exploits interspecific hybridization to combine vigour control, disease resistance and abiotic-stress tolerance (Gainza et al., 2015; Jiménez et al., 2013; Opazo et al., 2020). To capture a broad range of water-use strategies within a single screening experiment, we include different genotype pedigrees of Prunus rootstocks (**Table 1**), from commercial hybrids with well-characterized contrasting drought responses (ROOTPAC^®^40 and ROOTPAC^®^20; Opazo et al., 2019) to advanced interspecific breeding lines whose diurnal physiological behaviour has not yet been characterized.

**Table 1:** Pedigree of the species studied.

Rootstock	Pedigree
Rootpac^®^ 40	*P. dulcis* x *P. persica*
Rootpac^®^ 20	*P. besseyi* x *P. cerasifera*
Garnem GxN15	*P. dulcis* x *P. persica*
CEAF 176303	(*P. besseyi* x *P. cerasifera*) x *P. cerasifera, sub. Sp. Pissardii*
CEAF 197408	*P. cesarus* x *P. incisa*
CEAF 203001	*P. avium* x *P. cerasifera*

Spectral reflectance indices offer quick, non-destructive phenotyping and can help address the phenotyping bottleneck (Araus and Cairns, 2014; Lobos et al., 2022). The Photochemical Reflectance Index (PRI) was initially proposed as an indicator of short-term photosynthetic regulation (Gamon et al., 1992; Peñuelas et al., 1995) and has been linked to drought conditions (Thenot et al., 2002; Ripullone et al., 2011). However, under field conditions, PRI–radiation relationships can be unstable (Magney et al., 2016). Vapor pressure deficit (VPD) is a key factor influencing stomatal conductance, transpiration, and energy balance (Marino et al., 2014; Opazo et al., 2024), and previous research indicates that PRI can vary with VPD (Magney et al., 2016; D’Odorico et al., 2021). Although PRI has repeatedly been linked to VPD — as a driver of high-frequency PRI dynamics (Magney et al., 2016), in drone-based detection of tree drought responses (D’Odorico et al., 2021) and combined with VPD to estimate canopy light-use efficiency (Nakaji et al., 2014) — these approaches used VPD mainly to explain or correct PRI at the stand or canopy scale, not to derive comparable genotype descriptors.

We hypothesize that structuring diurnal PRI along the VPD gradient, and normalizing it to a common reference VPD, yields transferable components that allow genotypes to be compared across days of differing atmospheric demand. We therefore decompose the diurnal PRI signature into basal PRI at VPDref, the PRI–VPD slope, and WW–WD separation at VPDref, and test their temporal stability in six *Prunus* rootstock genotypes under well-watered and water-deficit conditions.

## 2 Materials and methods

The study was performed at CEAF (Rengo, Chile; 34°19’S, 70°50’W). One-year-old clonal plants were grown in 20-L pots (peat:perlite 1:1, v/v) in a randomized complete block design with four replicates per genotype × water-regime. We evaluated two commercial rootstocks with contrasting drought responses: ROOTPAC^®^40 (R40; drought-tolerant, and ROOTPAC^®^20 (R20; drought-sensitive) (Opazo et al., 2019; Toro et al., 2023; Opazo et al., 2024), and four breeding lines: GARNEM, CEAF197408, CEAF176303, and CEAF203001 (pedigree in **Table 1**). Field capacity was set gravimetrically, and pots were weighed daily. Well-watered plants were maintained near field capacity, while WD plants were maintained at 40% of field capacity. WD was imposed seven days before the first reflectance campaign and maintained for ten days.

Leaf reflectance (350–2,500 nm) was measured hourly (10:00–18:00) on February 9, 14, and 16, 2023, using a FieldSpec 4 Hi-Res spectroradiometer with contact probe illumination and frequent Spectralon calibration. Three fully expanded sun-exposed leaves per plant were averaged. PRI was computed as (R550 − R531)/(R550 + R531) (Gamon et al., 1992; Peñuelas et al., 1995). Meteorological data (i.e., radiation, ambient temperature and relative humidity) were obtained from an automated weather station; the three days spanned VPDmax ≈ 4.9, 3.6, and 3.3 kPa.

Gas exchange and leaf water potential were measured three healthy and fully expanded, sun-exposed leaf (near but different from leaf reflectance) per plant and water-regime treatment. Gas exchange was measured at solar noon on February 9 and 16 using a CIRAS-2 (25 °C, 40–50% RH, 400 μmol CO_2_ mol^−1^) to obtain stomatal conductance (gs), net CO_2_ assimilation (An), intercellular CO_2_ (Ci) and transpiration (E), and to compute intrinsic water-use efficiency (WUEi = An/gs). After gas exchange measurements, on the same leaves, midday water potential (LWP) was assessed using a pressure chamber (Model 1505D, PMS Instrument, Oregon, USA). WD plants averaged −1.63 MPa (Feb 9) and −1.62 MPa (Feb 16), while WW plants averaged −0.95 MPa and −0.84 MPa, respectively, indicating a moderate and stable deficit during measurements.

For each genotype, date, and water-regime, PRI was regressed against VPD (ordinary least squares). From fitted relationships, we derived three diurnal PRI signature components: (i) basal PRI at VPDref = 2.0 kPa, (ii) slope (ΔPRI/ΔVPD), and (iii) WW–WD separation at VPDref (difference between fitted WW and WD PRI at VPDref). Water-regime effects were tested using ANCOVA (PRI ~ VPD × water-regime). Stability across dates was assessed with Spearman rank correlations of genotype rankings for each component. PRI–physiology links were evaluated using genotype-level percentage changes (Δ%) between WD and WW for PRI and for each physiological variable. All analyses were performed using R 4.0.0 (R Core Team, 2021).

## 3 Results

There is a clear pattern of PRI activity throughout the day across different environments, and this pattern remains consistent from day to day (**Figure 1**). Across days and genotypes, PRI related weakly and inconsistently to incident radiation (data not shown) but formed clearer trajectories when plotted against VPD (**Figure 2**), improving discrimination among genotypes and water-regime (Magney et al., 2016; D’Odorico et al., 2021). ANCOVA showed that the water-regime main effect was usually significant, whereas VPD × water-regime interactions were uncommon, indicating that WD effects were mainly expressed as vertical shifts between WW and WD PRI–VPD trajectories rather than systematic slope changes (**Table 2**).

**Figure 1:**
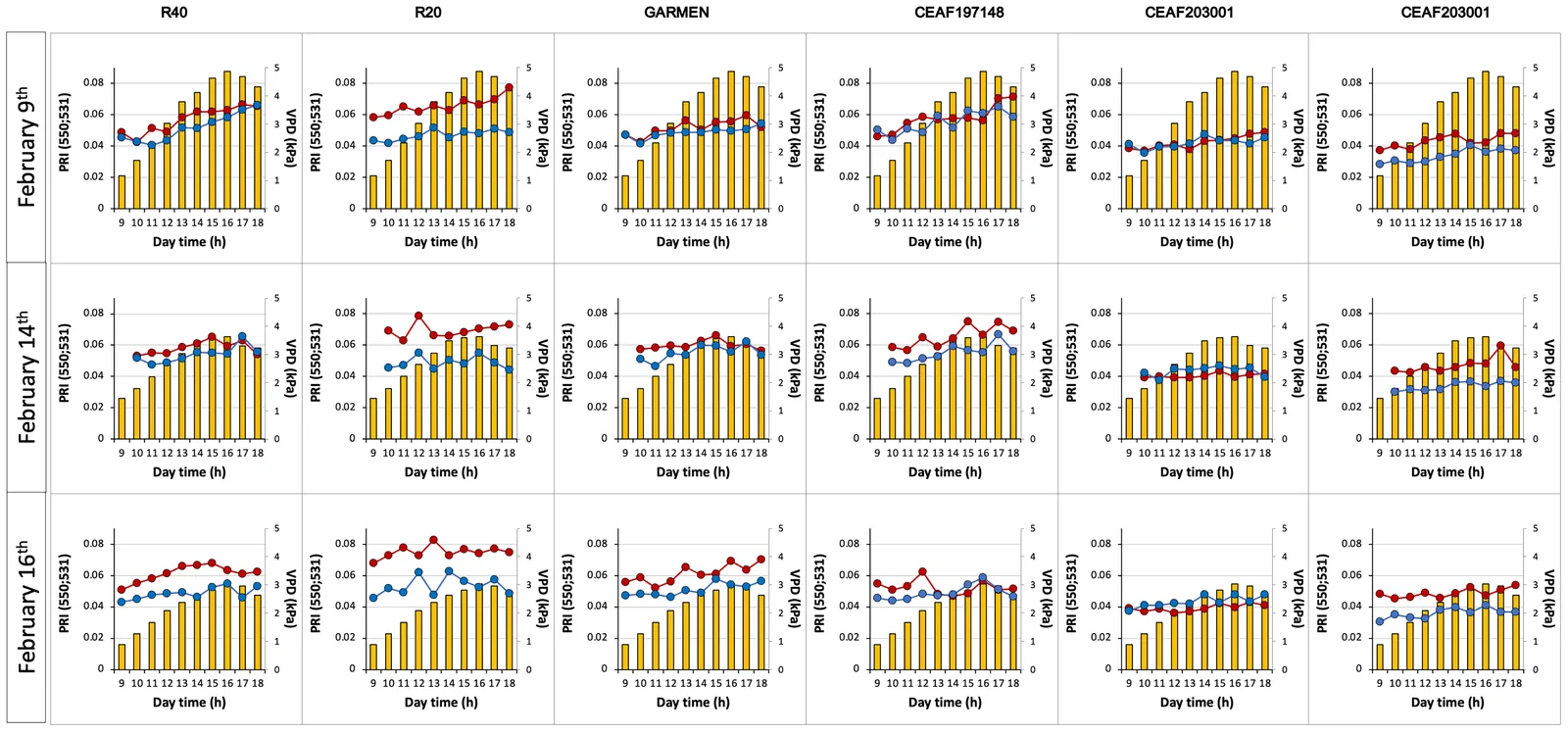
Daily changes in the Photochemical Reflectance Index (PRI) and vapor pressure deficit VPD for six *Prunus* rootstock genotypes under well-watered (WW, blue) and water-deficit (WD, red) conditions across three measurement dates. Hourly measurements taken from 10:00 to 18:00 h.

**Figure 2:**
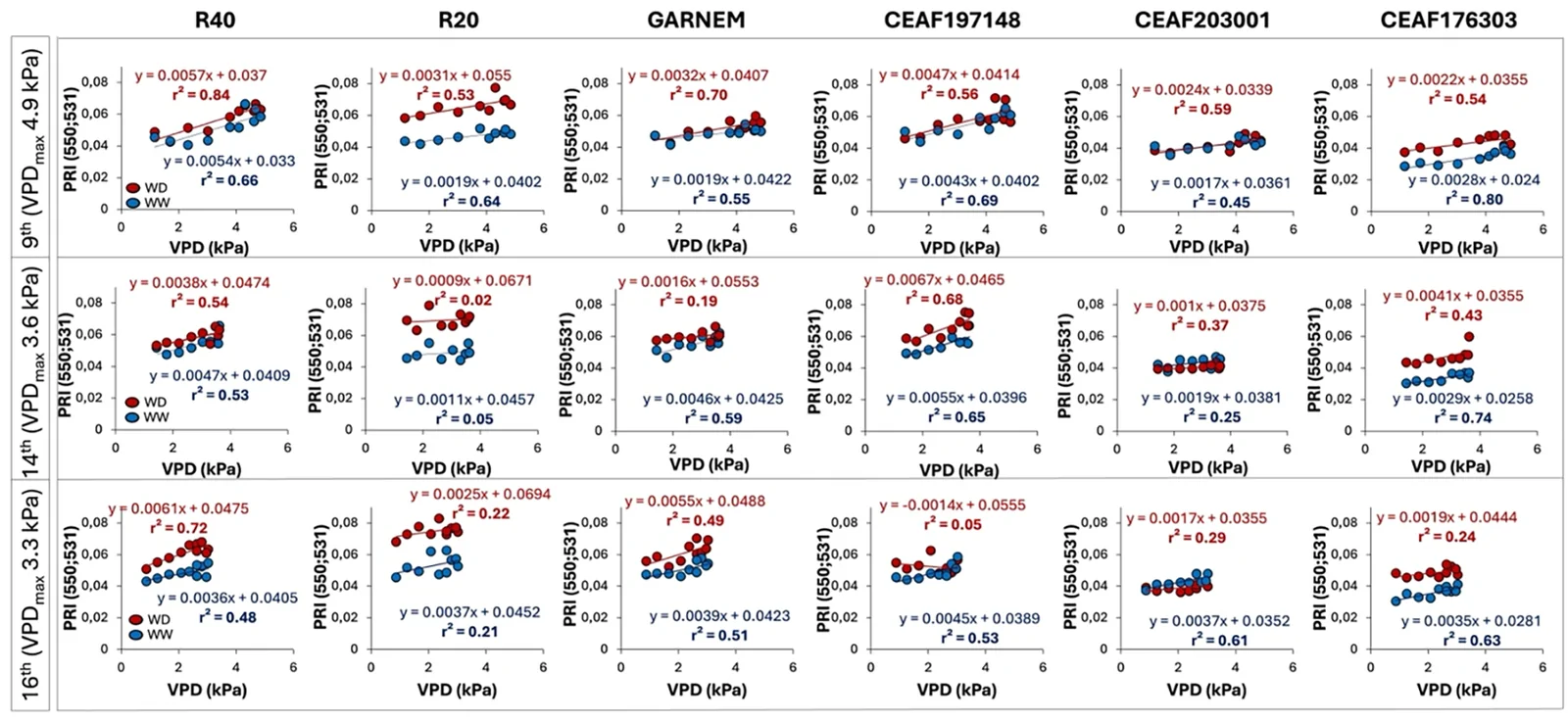
Diurnal patterns of the Photochemical Reflectance Index (PRI) and vapor pressure deficit (VPD) in six *Prunus* rootstock genotypes under well-watered (WW, blue) and water-deficit (WD, red) conditions across three measurement dates. Hourly measurements taken from 10:00 to 18:00 hours.

**Table 2:** Genotype-specific analysis of covariance (ANCOVA) of photochemical reflectance index (PRI) responses to vapor pressure deficit (VPD), water regime, and their interaction across three measurement dates.

Genotype	Date	N	d.f.	F VPD	p VPD	F at 2 kPa	p at 2 kPa	p regime Holm	F interaction	p interaction	p interaction Holm
R20	09-02	18	14	11.5380	4.341E-03	34.0865	4.305E-05	6.027E-04	0.2330	6.367E-01	1.000E+00
R40	09-02	18	14	56.5701	2.787E-06	1.9133	1.883E-01	7.530E-01	3.398E-06	9.986E-01	1.000E+00
Garmen	09-02	18	14	31.8486	6.061E-05	0.3409	5.686E-01	1.000E+00	1.2454	2.832E-01	1.000E+00
CEAF197148	09-02	18	14	19.8993	5.381E-04	1.3722	2.610E-01	7.829E-01	0.2663	6.139E-01	1.000E+00
CEAF176303	09-02	18	14	19.5767	5.769E-04	23.8432	2.419E-04	2.903E-03	0.9255	3.523E-01	1.000E+00
CEAF203001	09-02	18	14	20.8737	4.377E-04	0.0063	9.380E-01	1.000E+00	0.0748	7.884E-01	1.000E+00
R20	14-02	18	14	0.4755	5.017E-01	45.2587	9.665E-06	1.450E-04	0.0051	9.440E-01	1.000E+00
R40	14-02	18	14	15.8409	1.369E-03	4.2478	5.839E-02	3.503E-01	0.1549	6.998E-01	1.000E+00
Garmen	14-02	18	14	10.5266	5.877E-03	10.9332	5.194E-03	4.155E-02	2.3927	1.442E-01	1.000E+00
CEAF197148	14-02	18	14	27.6877	1.202E-04	12.9578	2.900E-03	2.610E-02	0.2591	6.187E-01	1.000E+00
CEAF176303	14-02	18	14	13.5098	2.495E-03	33.9519	4.393E-05	6.027E-04	0.4443	5.159E-01	1.000E+00
CEAF203001	14-02	18	14	4.7671	4.653E-02	2.3832	1.449E-01	7.247E-01	0.3857	5.446E-01	1.000E+00
R20	16-02	18	14	0.5165	4.842E-01	64.6045	1.296E-06	2.204E-05	0.1549	6.998E-01	1.000E+00
R40	16-02	18	14	10.7050	5.565E-03	51.1765	4.906E-06	7.850E-05	0.5027	4.899E-01	1.000E+00
Garmen	16-02	18	14	11.9622	3.838E-03	18.5639	7.215E-04	7.936E-03	0.3382	5.701E-01	1.000E+00
CEAF197148	16-02	18	14	1.8585	1.943E-01	5.4005	3.569E-02	2.499E-01	3.9748	6.604E-02	1.000E+00
CEAF176303	16-02	18	14	8.9631	9.669E-03	76.8396	4.652E-07	8.373E-06	0.0096	9.235E-01	1.000E+00
CEAF203001	16-02	18	14	10.8742	5.287E-03	13.8305	2.290E-03	2.290E-02	0.0382	8.479E-01	1.000E+00

Models were fitted separately for each genotype and measurement date using hourly observations from 10:00 to 18:00 h. Water-regime effects test the difference between water-deficit (WD) and well-watered (WW) conditions at VPD = 2.0 kPa. The VPD × water regime interaction tests differences in PRI–VPD slopes.

The decomposition revealed consistent genotypic variability. Basal PRI at VPDref was lowest in CEAF203001 and CEAF176303 and higher in R40, R20, GARNEM, and CEAF197148 (**Table 3**). Slopes varied among genotypes and tended to be larger under WD than under WW, indicating greater diurnal adjustment under deficit conditions. WW–WD separation at VPDref captured genotype-dependent water-regime differences, where CEAF203001 showed minimal separation (near zero), whereas R20 displayed strong and consistent separation across measurement days.

**Table 3:** Quantitative decomposition of the diurnal PRI (550;531) signature at VPDref = 2.0 kPa with qualitative ranking across genotypes.

Genotype	Basal PRI		Slope		WW–WD
	WW	WD	WW	WD	separation
R20	0.0480 ± 0.0042	0.0682 ± 0.0067	0.0023 ± 0.0012	0.0021 ± 0.0012	0.0201 ± 0.0027
	**medium**	**high**	**low**	**low**	**high**
R40	0.0476 ± 0.0036	0.0545 ± 0.0057	0.0043 ± 0.0010	0.0052 ± 0.0012	0.0069 ± 0.0045
	**medium**	**medium**	**high**	**high**	**medium**
GARNEM	0.0493 ± 0.0031	0.0552 ± 0.0070	0.0033 ± 0.0013	0.0035 ± 0.0020	0.0058 ± 0.0044
	**high**	**medium**	**medium**	**medium**	**medium**
CEAF197148	0.0492 ± 0.0017	0.0547 ± 0.0051	0.0047 ± 0.0005	0.0032 ± 0.0041	0.0055 ± 0.0038
	**high**	**medium**	**high**	**medium**	**medium**
CEAF176303	0.0321 ± 0.0028	0.0441 ± 0.0041	0.0030 ± 0.0004	0.0026 ± 0.0010	0.0120 ± 0.0015
	**low**	**low**	**medium**	**medium**	**high**
CEAF203001	0.0413 ± 0.0017	0.0391 ± 0.0005	0.0023 ± 0.0012	0.0017 ± 0.0007	−0.0023 ± 0.0015
	**low**	**low**	**low**	**low**	**low**

Qualitative ranks (high, medium, low) indicate the relative position of each genotype within the experimental panel (n = 6) for each PRI signature component and are intended solely to facilitate comparative interpretation.

Despite changing atmospheric demand (VPDmax from ≈4.9 to ≈3.3 kPa), genotype rankings for basal PRI WD and WW–WD separation were relatively stable across dates (Spearman ρ ≈ 0.771–0.943 and 0.600–0.943), while basal PRI WW and slope rankings were less stable (**Table 4**). Genotype-level ΔPRI aligned with shifts in midday physiology, correlating most strongly with Δgs and ΔE (**Figure 3**), and more moderately with ΔAn; associations with ΔCi and ΔLWP were negative (Marino et al., 2014; Zhang et al., 2017b).

**Table 4:** Spearman rank correlations between measurement dates for diurnal photochemical reflectance index (PRI) signature components across six *Prunus* rootstock genotypes.

Componente	Date 1 *vs*. 2	Date 1 *vs*. 3	Date 2 *vs*. 3
Basal PRI WW	0.886 (*p* = 0.0333)	0.600 (*p* = 0.2417)	0.600 (*p* = 0.2417)
Basal PRI WD	0.943 (*p* = 0.0167)	0.829 (*p* = 0.0583)	0.771 (*p* = 0.1028)
Slope WW	0.886 (*p* = 0.0333)	0.600 (*p* = 0.2417)	0.886 (*p* = 0.0333)
Slope WD	0.257 (*p* = 0.6583)	0.086 (*p* = 0.9194)	−0.086 (*p* = 0.9194)
WD − WW separation a 2,0 kPa	0.943 (*p* = 0.0167)	0.714 (*p* = 0.1361)	0.600 (*p* = 0.2417)

Values are Spearman’s rank correlation coefficients (ρ), with exact two-sided *p*-values in parentheses (*n* = 6 genotypes). Correlations compare genotype rankings between February 9, 14, and 16, 2023, using components estimated from 10:00–18:00 h observations. Basal PRI and treatment separation (WD − WW) were evaluated at a reference vapor pressure deficit of 2.0 kPa. WW, well-watered; WD, water-deficit.

**Figure 3:**
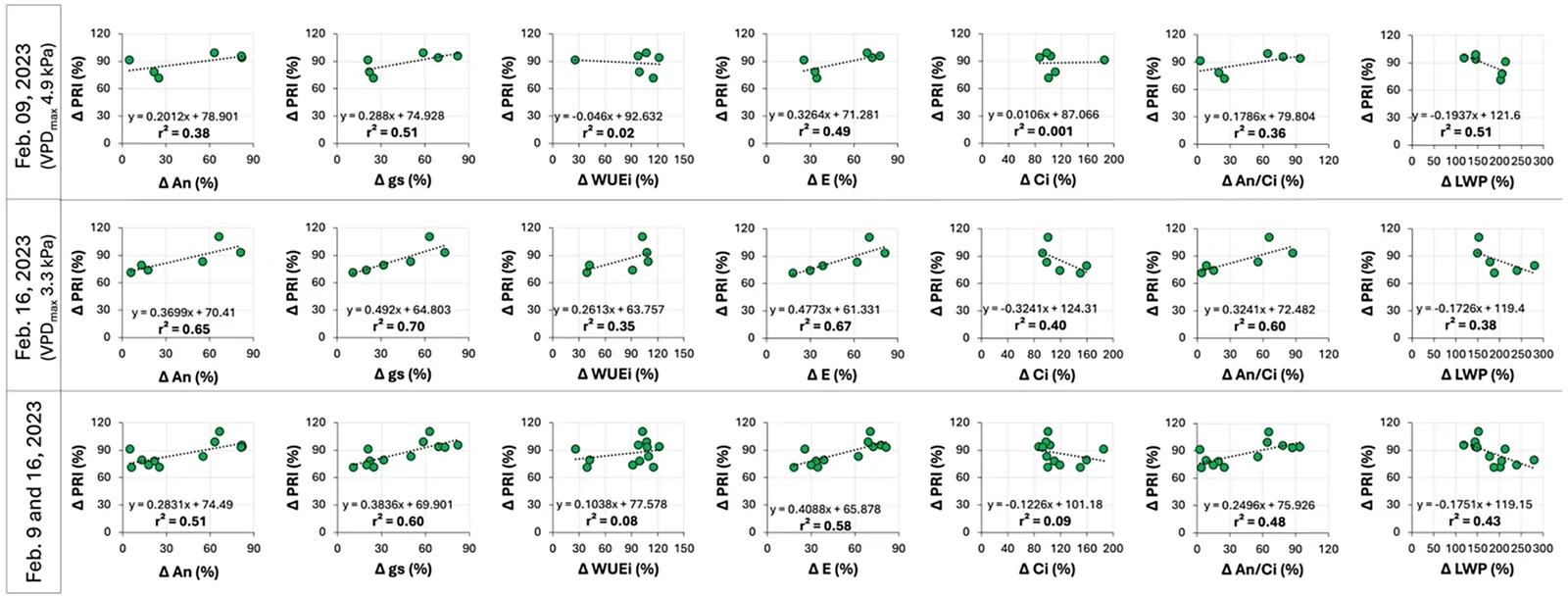
Genotype-level relationships between changes in PRI (ΔPRI) and alterations in physiological variables (ΔAn, Δgs, ΔWUEi, ΔE, ΔCi, ΔAn/Ci, and ΔLWP) under water deficit compared to well-watered conditions on two measurement dates and when combined. Each point indicates a genotype-level percentage change (Δ%) between WW and WD conditions, not a sequence over time within individual plants.

## 4 Discussion and conclusions

We present a field-ready method that uses VPD (computed from routine temperature and humidity data) to structure diurnal PRI and extract repeatable components. This approach complements single-time-point screening by separating (i) baseline PRI under low demand, (ii) sensitivity to increasing demand, and (iii) water-regime effects expressed as WW–WD separation. In our *Prunus* panel, basal PRI and WW–WD separation were repeatable across days with contrasting VPDmax, satisfying a key requirement for breeding traits (i.e, stability under unavoidable environmental variability).

Mechanistically, PRI tracks the epoxidation state of the xanthophyll cycle: under excess absorbed radiation, violaxanthin is de-epoxidized to zeaxanthin, driving reflectance changes near 531 nm and reflecting thermal (non-photochemical) dissipation of excess energy (Gamon et al., 1992; Peñuelas et al., 1995). Because the size of the xanthophyll pool and the capacity for thermal dissipation are partly genotype-dependent, basal PRI and its diurnal rise under increasing demand can be read as genotype-specific descriptors of photoprotective capacity, which is consistent with the stable genotype rankings we observed for basal PRI.

The strength of instantaneous PRI–physiology couplings was context-dependent, being weaker on the highest-demand day (VPDmax ≈ 4.9 kPa) and stronger on lower-demand days. This is consistent with a VPD threshold above which stomata approach their regulatory limit and further increases in evaporative demand no longer translate into proportional changes in PRI, so that at very high VPD several genotypes converge and genotypic contrasts are compressed (Marino et al., 2014; Zhang et al., 2017b).

Because PRI integrates fast photosynthetic regulation (Gamon et al., 1992; Peñuelas et al., 1995) and responds to multiple drivers (Rebetzke et al., 2012; Zhang et al., 2017a; Yang et al., 2020), we treat WW–WD separation as a discriminatory feature rather than a direct proxy for drought tolerance. Because VPD, incident radiation and time of day co-vary during clear days, we deliberately used VPD as a single, physiologically grounded organizing variable rather than a multivariable predictor; VPD outperformed radiation in structuring diurnal PRI. Our conclusions are nonetheless based on leaf-level measurements, in pots, under moderate and stable water deficit (WD ≈ −1.6 MPa vs. WW ≈ −0.9 MPa), across four replicates and three days. While the agreement of genotype rankings across days of contrasting VPDmax supports the repeatability of basal PRI and WW–WD separation, independent validation against agronomic performance, and across seasons, developmental stages and canopy scales (ground platforms or drones), remains necessary before these descriptors can be used as selection criteria (Suárez et al., 2008; Zarco-Tejada et al., 2013).

Future work should integrate complementary optical measurements (pigments, fluorescence) to strengthen mechanistic interpretation (Estrada et al., 2023). Nonetheless, structuring diurnal PRI along VPD gradients provides a simple, scalable way to quantify genotype-specific PRI signatures and to compare different genetics under water deficit in Mediterranean field phenotyping contexts.

## Data Availability

The raw data supporting the conclusions of this article will be made available by the authors, without undue reservation.

## References

[B1] Araus J. L. Cairns J. E. (2014). Field high-throughput phenotyping: the new crop breeding frontier. Trends Plant Sci. 19, 52–61. doi: 10.1016/j.tplants.2013.09.008 24139902

[B2] D’Odorico P. Schönbeck L. Vitali V. Meusburger K. Schaub M. Ginzler C. . (2021). Drone-based physiological index reveals long-term acclimation and drought stress responses in trees. Plant Cell Environ. 44, 3552–3570. doi: 10.1111/pce.14177 34462922 PMC9292485

[B3] Estrada F. Flexas J. Araus J. L. Mora-Poblete F. Gonzalez-Talice J. Castillo D. . (2023). Exploring plant responses to abiotic stress by contrasting spectral signature changes. Front. Plant Sci. 13, 1026323. doi: 10.3389/fpls.2022.1026323 36777544 PMC9910286

[B4] Gainza F. Opazo I. Guajardo V. Meza P. Ortiz M. Pinochet J. . (2015). Rootstock breeding in *Prunus* species: ongoing efforts and new challenges. Chil. J. Agric. Res. 75, 6–16. doi: 10.4067/S0718-58392015000300002

[B5] Gamon J. A. Peñuelas J. Field C. B. (1992). A narrow-waveband spectral index that tracks diurnal changes in photosynthetic efficiency. Remote Sens. Environ. 41, 35–44. doi: 10.1016/0034-4257(92)90059-S

[B6] Hancock J. F. Scorza R. Lobos G. A. (2008). “ Peaches,” in Temperate Fruit Crop Breeding: Germplasm to Genomics. Ed. Hancock J. F. ( Springer, Dordrecht), 265–298. doi: 10.1007/978-1-4020-6907-9_9

[B7] Jiménez S. Dridi J. Gutiérrez D. Moret D. Irigoyen J. J. Moreno M. A. . (2013). Physiological, biochemical and molecular responses in four *Prunus* rootstocks submitted to drought stress. Tree Physiol. 33, 1061–1075. doi: 10.1093/treephys/tpt074 24162335

[B8] Leakey A. D. B. Ferguson J. N. Pignon C. P. Wu A. Jin Z. Hammer G. L. . (2019). Water use efficiency as a constraint and target for improving the resilience and productivity of C3 and C4 crops. Annu. Rev. Plant Biol. 70, 781–808. doi: 10.1146/annurev-arplant-042817-040305 31035829

[B9] Lobos G. A. Estrada F. del Pozo A. Romero-Bravo S. Astudillo C. A. Mora-Poblete F. (2022). “ Challenges for a massive implementation of phenomics in plant breeding programs,” in High-Throughput Plant Phenotyping: Methods and Protocols. Eds. Lorence A. Medina Jimenez K. ( Springer, New York), 135–157. doi: 10.1007/978-1-0716-2537-8_13 35895202

[B10] Magney T. S. Vierling L. A. Eitel J. U. H. Huggins D. R. Garrity S. R. (2016). Response of high frequency photochemical reflectance index (PRI) measurements to environmental conditions in wheat. Remote Sens. Environ. 173, 84–97. doi: 10.1016/j.rse.2015.11.01342574925

[B11] Marino G. Pallozzi E. Cocozza C. Tognetti R. Giovannelli A. Cantini C. . (2014). Assessing gas exchange, sap flow and water relations using tree canopy spectral reflectance indices in irrigated and rainfed *Olea europaea* L. Environ. Exp. Bot. 99, 43–52. doi: 10.1016/j.envexpbot.2013.10.00842574925

[B12] Medrano H. Tortosa I. Montes E. Pou A. Balda P. Bota J. . (2018). “ Genetic improvement of grapevine (*Vitis vinifera* L.) water use efficiency: variability among varieties and clones,” in Water Scarcity and Sustainable Agriculture in Semiarid Environment. Eds. García Tejero I. F. Durán Zuazo V. H. (United States: Academic Press), 377–401. doi: 10.1016/B978-0-12-813164-0.00016-8

[B13] Nakaji T. Kosugi Y. Takanashi S. Niiyama K. Noguchi S. Tani M. Oguma H. Nik AR Kassim AR . (2014). Estimation of light-use efficiency through a combinational use of the photochemical reflectance index and vapor pressure deficit in an evergreen tropical rainforest at Pasoh, Peninsular Malaysia. Remote Sens Environ. 150, 80–92. doi: 10.1016/j.rse.2014.04.021

[B14] Opazo I. Pimentel P. Salvatierra A. Ortiz M. Toro G. Garrido-Salinas M. (2024). Water stress tolerance is coordinated with water use capacity and growth under water deficit across six fruit tree species. Irrig. Sci. 42, 493–507. doi: 10.1007/s00271-024-00915-930311153

[B15] Opazo I. Toro G. Salvatierra A. Pastenes C. Pimentel P. (2020). Rootstocks modulate the physiology and growth responses to water deficit and long-term recovery in grafted stone fruit trees. Agric. Water Manage. 228, 105897. doi: 10.1016/j.agwat.2019.10589742574925

[B16] Opazo I. Toro G. Solis S. Salvatierra A. Franck N. Albornoz F. . (2019). Late reduction on transpiration is an important trait for water deficit tolerance in interspecific *Prunus* rootstock hybrids. Theor. Exp. Plant Physiol. 31, 493–506. doi: 10.1007/s40626-019-00162-w30311153

[B17] Pareek A. Dhankher O. P. Foyer C. H. (2020). Mitigating the impact of climate change on plant productivity and ecosystem sustainability. J. Exp. Bot. 71, 451–456. doi: 10.1093/jxb/erz518 31909813 PMC6945998

[B18] Peñuelas J. Filella I. Gamon J. A. (1995). Assessment of photosynthetic radiation-use efficiency with spectral reflectance. New Phytol. 131, 291–296. doi: 10.1111/j.1469-8137.1995.tb03064.x40046247

[B19] R Core Team (2021). R: A Language and Environment for Statistical Computing (Vienna, Austria: R Foundation for Statistical Computing). Available online at: https://www.R-project.org/ (Accessed May 3, 2026).

[B20] Rebetzke G. J. Chenu K. Biddulph B. Moeller C. Deery D. M. Rattey A. R. . (2012). A multisite managed environment facility for targeted trait and germplasm phenotyping. Funct. Plant Biol. 40, 1–13. doi: 10.1071/FP12180 32481082

[B21] Ripullone F. Rivelli A. R. Baraldi R. Guarini R. Guerrieri R. Magnani F. . (2011). Effectiveness of the photochemical reflectance index to track photosynthetic activity over a range of forest tree species and plant water statuses. Funct. Plant Biol. 38, 177–186. doi: 10.1071/FP10078 32480874

[B22] Suárez L. Zarco-Tejada P. J. Sepulcre-Cantó G. Pérez-Priego O. Miller J. R. Jiménez-Muñoz J. C. . (2008). Assessing canopy PRI for water stress detection with diurnal airborne imagery. Remote Sens. Environ. 112, 560–575. doi: 10.1016/j.rse.2007.05.00942574925

[B23] Thenot F. Méthy M. Winkel T. (2002). The photochemical reflectance index (PRI) as a water-stress index. Int. J. Remote Sens. 23, 5135–5149. doi: 10.1080/0143116021016310037339054

[B24] Toro G. Pastenes C. Salvatierra A. Pimentel P. (2023). Trade-off between hydraulic sensitivity, root hydraulic conductivity and water use efficiency in grafted *Prunus* under water deficit. Agric. Water Manage. 282, 108284. doi: 10.1016/j.agwat.2023.10828442574925

[B25] Vogel E. Donat M. G. Alexander L. V. Meinshausen M. Ray D. K. Karoly D. . (2019). The effects of climate extremes on global agricultural yields. Environ. Res. Lett. 14, 054010. doi: 10.1088/1748-9326/ab154b

[B26] Yang J. C. Magney T. S. Yan D. Knowles J. F. Smith W. K. Scott R. L. Barron‐Gafford G. A. (2020). The photochemical reflectance index (PRI) captures the ecohydrologic sensitivity of a semiarid mixed conifer forest. J. Geophysical Res.: Biogeosci. 125, e2019JG005624. doi: 10.1029/2019JG00562429143083

[B27] Zarco-Tejada P. J. González-Dugo V. Williams L. E. Suárez L. Berni J. A. J. Goldhamer D. . (2013). A PRI-based water stress index combining structural and chlorophyll effects: assessment using diurnal narrow-band airborne imagery and the CWSI thermal index. Remote Sens. Environ. 138, 38–50. doi: 10.1016/j.rse.2013.07.02442574925

[B28] Zhang C. Filella I. Liu D. Ogaya R. Llusià J. Asensio D. . (2017a). Photochemical reflectance index (PRI) for detecting responses of diurnal and seasonal photosynthetic activity to experimental drought and warming in a Mediterranean shrubland. Remote Sens. 9, 1189. doi: 10.3390/rs911118930654563

[B29] Zhang C. Preece C. Filella I. Farré-Armengol G. Peñuelas J. (2017b). Assessment of the response of photosynthetic activity of Mediterranean evergreen oaks to enhanced drought stress and recovery using PRI and R690/R630. Forests 8, 386. doi: 10.3390/f810038630654563

